# Selective denervation of the corrugator supercilii muscle for the treatment of idiopatic trigeminal neuralgia purely paroxysmal distributed in the supraorbital and suprathrochlear dermatomes

**DOI:** 10.1186/s10194-021-01218-6

**Published:** 2021-03-04

**Authors:** Alessandro Gualdi, Janos Cambiaso-Daniel, Jonatann Gatti, Ziv M. Peled, Robert Hagan, Dario Bertossi, Paul Wurzer, Lars-Peter Kamolz, Saja Scherer, Giorgio Pietramaggiori

**Affiliations:** 1 University Vita-Salute San Raffaele, Milan, Italy; 2 Surgical Medical Group, Milan, Italy; 3 Division of Plastic, Aesthetic and Reconstructive Surgery, Department of Surgery, Medical University of Graz, Graz, Austria; 4 Peled Plastic Surgery, San Francisco, California USA; 5 Neuropax Clinic, St. Louis, MO USA; 6 Maxillo Facial, Plastic Surgery Unit, Department of Surgery, Policlinico G.B. Rossi, University of Verona, Verona, Italy; 7 Clinical Professor, Centre for Integrated Medical and Translational Research, University of London, London, UK; 8 Research Unit Safety in Health, Division of Plastic, Aesthetic and Reconstructive Surgery, Department of Surgery, Medical University of Graz, Graz, Austria; 9 Global Medical Institute and Swiss Nerve Center, Lausanne, Switzerland; 10 Plastic and Reconstructive Surgery, Department of Neurosciences, University of Padua, Padua, Italy

**Keywords:** Migraine disorders, Headache disorders, Quality of life, Neuralgia, Botulinum toxin type a

## Abstract

**Introduction:**

Idiopatic trigeminal neuralgia purely paroxysmal (ITNp) distributed in the supraorbital and suprathrochlear dermatomes (SSd), refractory to conventional treatments have been linked to the hyperactivity of the corrugator supercilii muscle (CSM). In these patients, the inactivation of the CSM via botulinum toxin type A (BTA) injections has been proven to be safe and effective in reducing migraine burden. The main limitation of BTA is the need of repetitive injections and relative high costs. Based on the study of the motor innervation of the CSM, we describe here an alternative approach to improve these type of migraines, based on a minimally invasive denervation of the CSM.

**Materials and methods:**

Motor innervation and feasibility of selective CSM denervation was first studied on fresh frozen cadavers. Once the technique was safely established, 15 patients were enrolled. To be considered eligible, patients had to meet the following criteria: positive response to BTA treatment, migraine disability assessment score > 24, > 15 migraine days/month, no occipital/temporal trigger points and plausible reasons to discontinue BTA treatment. Pre- and post- operative migraine headache index (MHI) were compared, and complications were classified following the Clavien-Dindo classification (CDC).

**Results:**

Fifteen patients (9 females and 6 males) underwent the described surgical procedure. The mean age was 41 ± 10 years. Migraine headache episodes decreased from 24 ± 4 day/month to 2 ± 2 (*p* < 0.001) The MHI decreased from 208 ± 35 to 10 ± 11 (*p* < 0.001). One patient (7%) had a grade I complication according to the CDC. No patient needed a second operative procedure.

**Conclusions:**

Our findings suggest that the selective CSM denervation represents a safe and minimally invasive approach to improve ITNp distributed in the SSd associated with CSM hyperactivation.

**Trial registration:**

The data collection was conducted as a retrospective quality assessment study and all procedures were performed in accordance with the ethical standards of the national research committee and the 1964 Helsinki Declaration and its later amendments.

**Supplementary Information:**

The online version contains supplementary material available at 10.1186/s10194-021-01218-6.

## Introduction

The number of patients affected by chronic headaches and migraines are increasing worldwide, as are direct and indirect costs for the social and healthcare systems. This disability can dramatically impact patients’ quality of life with negative effects comparable to patients with chronic diseases as diabetes, dementia, quadriplegia and active psychosis [1–3].

The corrugator supercilii muscle (CSM), along with the procerus and the depressor supercilii muscles, are medial eyebrow depressors and their contraction is associated with expressions of anger and displeasure [4]. As suggested by Guyuron et al., the hyperactivity of the CSM may play a role in the development of idiopatic trigeminal neuralgia purely paroxysmal (ITNp), characterized by pain in the territories of innervation of the supraorbital and supratrochlear nerve [5, 6]. The CSM receives a medial and lateral motor innervation from the facial nerve. The lateral innervation is provided by a temporal branch running 15 to 32 mm above the lateral orbital margin toward the midpoint of the lateral dermal insertion of the CSM [7, 8]. While the medial innervation is provided by small branches from the zygomatic, buccal or bucco-zygomatic branches of the facial nerve, running lateral to the angular vessels and reaching the medial insertion of the CSM 4 to 7 mm medial to the medial canthus [9, 10].

This hypothesis is supported by the efficacy of CSM inhibition via botulinum toxin type A (BTA) injections to decrease the headache burden [11, 12]. Since BTA was first shown to result in significant, but temporary improvement in ITNp distributed in the supraorbital and suprathrochlear dermatomes (SSd), a completely new field has evolved aiming to develop surgical techniques to obtain permanent improvements. Specifically, Dr. Guyuron observed that patients suffering from this type of headaches after forehead rejuvenation procedures that included CSM resection, exhibited a significant improvement in pain frequency and intensity [6]. He hypothesized supra-orbital neurovascular pedicle decompression was the reason behind the unexpected positive results on headaches [6, 13].

Over the ensuing 20 years, sensory nerves and vessels have been the object of many studies aiming to understand and improve ITNp distributed in the SSD, focusing on the anatomy and interactions of the CSM with the supra-orbital as well as supra-trochlear neurovascular bundles. Compression points along the course of these sensory nerves and/or mechanical nerve irritation via CSM contractions were identified as the main contributing factors (triggers) in the development of ITNp [7–10, 14–17].

Non-surgical treatments such as BTA injections into the CSM, and surgical approaches to decompress the supra-orbital neurovascular bundles, were described and proved to be effective in decreasing this type of headache also called supraorbital rim syndrome in over 80% of the treated cases [18–20]. In addition, several studies described the interaction between the CSM and the supraorbital nerve (SON) and supratrochlear nerve (STN) [15, 21]. However, only a few reports focused on the motor innervation of the CSM, which is a plausible alternative target for decreasing the burden of ITNp distributed in the SSd based on the efficacy of BTA treatment.

Considering the consistent innervation of the CSM and the positive effects of BTA injections on ITNp mainly distributed in the SSd, the aim of this study was to present a novel surgical CSM denervation technique, as a minimally invasive procedure.

## Material and methods

**Fig. 1:**
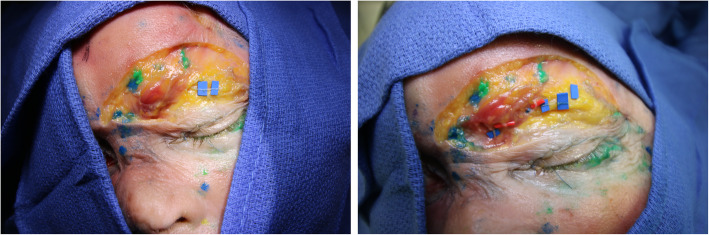
Anatomic dissection. After skin excision, the corrugator muscle (CSM) is identified. The location of subcutaneous, lateral attachments of the CSM have been marked in green via a transcutaneous injection. Sensitive branches from the supraorbital and supratrochlear nerves crossing the corrugator muscle (red marks) with a vertical trajectory are identified. Motor branches, parallel to the CSM are highlighted in blue

The technique was first performed on five fresh, Caucasian head specimens at the Institute of Anatomy of the Medical University of Lausanne in Switzerland. Both medial and lateral motor branches of the CSM, as described in literature, were identified (Fig. 1).

### Patient selection

Once all patients were diagnosed with chronic, refractory migraines by a neurologist and failed to respond to at least two disease modifying or preventative treatments, such as antidepressants, anti-epileptics and/or beta-blockers these were considered eligible for the selective CSM denervation procedure. In addition, the patients had to satisfy all of the following criteria:

- age > 18 years
- failure with migraine pharmacologic therapy or excessive intake (over and above the recommended safety limit)
- positive history and symptoms compatible with ITNp distributed in the SSd
- positive response (close to 100% symptom reduction) to three BTA treatments of the CSM as described by Guyuron et al. [11, 22].
- migraine disability assessment score (MIDAS) > 24
- no occipital/temporal trigger points

Furthermore, all candidates were unwilling to continue BTA treatment and preferred a permanent solution accepting the risk/benefits of the selective CSM denervation technique.

The data collection was conducted as a retrospective quality assessment study and all procedures were performed in accordance with the ethical standards of the national research committee and the 1964 Helsinki Declaration and its later amendments.

**Fig. 2:**
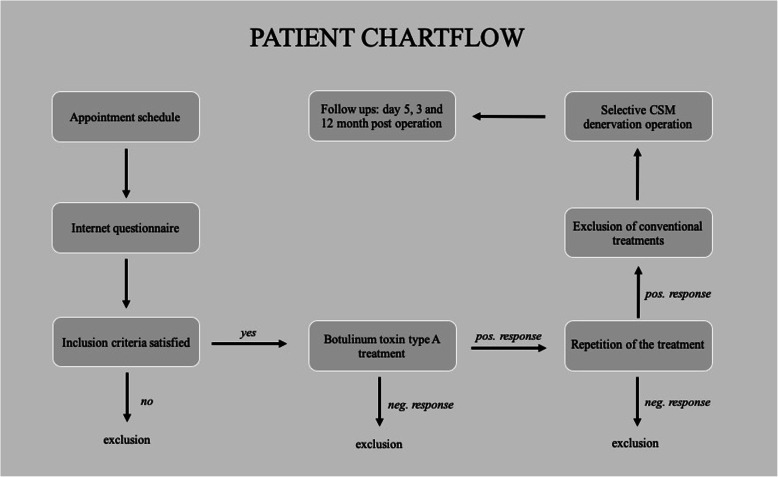
Patient flowchart. After failing to respond to conventional migraine treatments, patients were selected based on a positive (close to 100%) response to BTA injections. BTA injection were repeated 3 times in these patients and consistently achieved near to 100% remission of supraorbital headaches

A detailed patient flowchart is presented in Fig. 2.

### Surgical technique

**Fig. 3:**
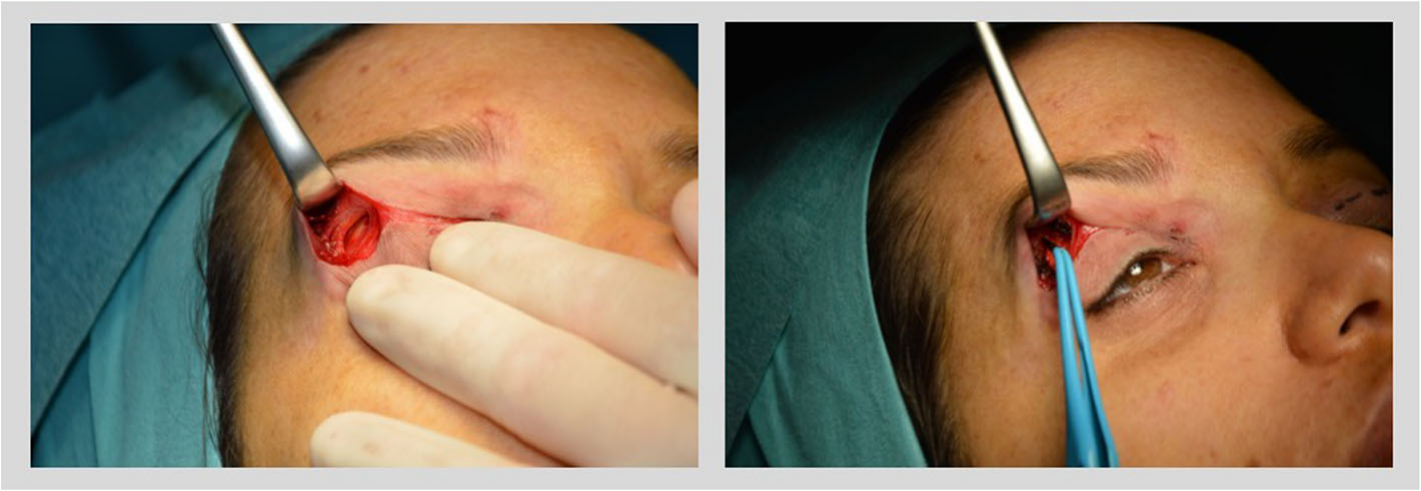
Lateral CSM motor innervation resection. Lateral to the lateral, subcutaneous insertion of the CSM, a tunnel was dissected in the preseptal space between superficial temporal fascia and the subcutaneous tissue. In this space, motor fibers were cauterized via a bipolar coagulation

**Fig. 4:**
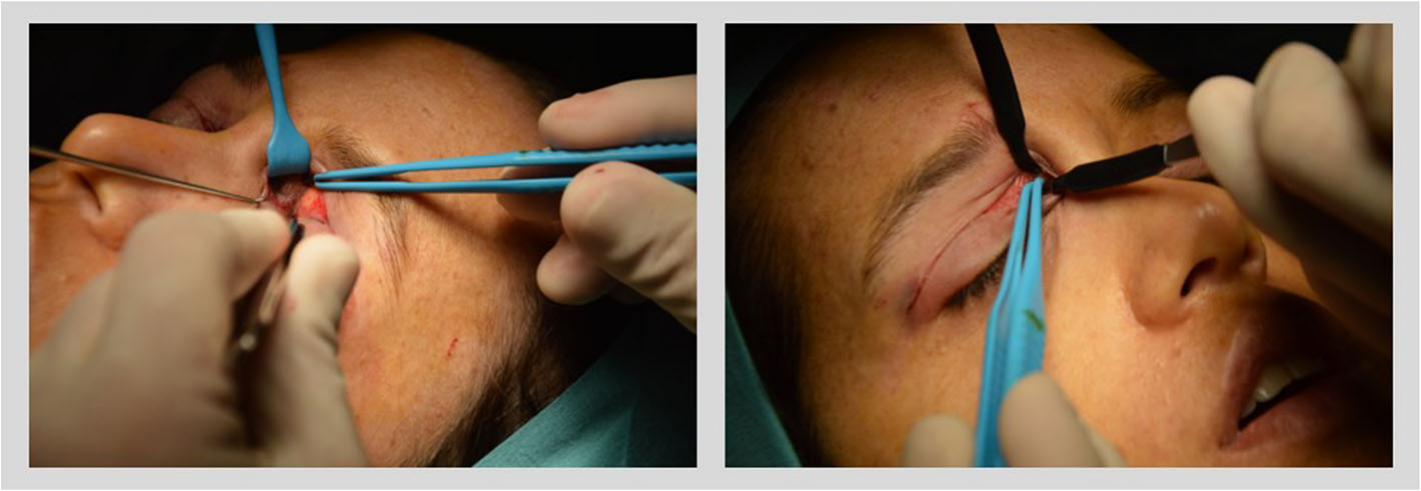
Medial CSM motor innervation resection. Medial to the medial insertion of the CSM and the supratrochlear pedicle, dissection was carried out to identify the medial motor inervation of the CSM. Medial motor branches to the CSM were dissected and cauterized via bipolar coagulation

Pre-operatively, patients were asked to frown so that the lateral and medial ends of the CSM were identified and marked on the skin. An incision line was marked along the superior palpebral crease from the medial to the lateral canthus with the patient in upstanding position.Each patient received then 0.5 mg/kg i.v. Diazepam as premedication. Tumescent anesthesia was infiltrated using a 30 Gauge needle (solution composition: 50% lidocaine 1% with 1:100000 epinephrine and 50% chirocaine 0.5%). Fifteen minutes after tumescent infiltration, the trans palpebral incision was carried out with a # 15 blade. Dissection was carried out with Stevens scissors to create a tunnel above and below the lateral and medial insertion of the CSM moving upward in the pre-septal space. Attention was paid not to dissect and damage any sensory nerve branches (particularly on the medial insertion of the CSM). In the central portion of the incision, no further dissection was carried out. Once the CSM muscle was isolated medially and laterally, the skin was retracted upwards and the denervation of the CSM was performed through bipolar coagulation (Figs. 3, 4) [7, 8, 10]. In the lateral part of the CSM attention was made not to extend the cauterization zone above the level of the corrugator, not to cause any injury to the frontal branches of the facial nerve going to the frontal muscle. The skin was repaired with a continuous intracutaneous nylon running suture (Prolene 5/0, Ethicon Inc., Somerville, NJ, USA), and Steri-Strips (3 M, Maplewood, MN, USA). Steri-Strips and sutures were removed 5 days after surgery.

The entire procedure is demonstrated in Video 1.


**Additional file 1: Video 1.** Surgical procedure. The main critical steps of the CSM denervation technique are described.

### Data collection

All patients were operated by the same surgical team in an outpatient setting between November 2015 and May 2019. Prior to any intervention, the migraine headache index (MHI) was calculated, trigger points were identified and demographics collected. Additionally, pictures and informed consent for the procedure were obtained. The postoperative complications were recorded and classified using the Clavien-Dindo classification (Table 1) [23]. Each patient underwent a follow up visit at 5 days, 3 and 12 months following the operation. At the 12-month visit, the MHI was calculated, and follow-up pictures were taken.

**Table 1:** The Clavien-Dindo classification [23]

Grade	Definition
Grade I	Any deviation from the normal postoperative course without the need for pharmacological treatment or surgical, endoscopic, and radiological interventions Allowed therapeutic regimens are: drugs as antiemetics, antipyretics, analgesics, diuretics, electrolytes, and physiotherapy. This grade also includes wound infections opened at the bedside
Grade II	Requiring pharmacological treatment with drugs other than such allowed for grade I complications Blood transfusions and total parenteral nutrition are also included
Grade III	Requiring surgical, endoscopic or radiological intervention
Grade IIIa	Intervention not under general anesthesia
Grade IIIb	Intervention under general anesthesia
Grade IV	Life-threatening complication (including CNS complications)^a^ requiring IC/ICU management
Grade IVa	Single organ dysfunction (including dialysis)
Grade IVb	Multiorgan dysfunction
Grade V	Death of a patient
Suffix “d”	If the patient suffers from a complication at the time of discharge the suffix “d” (for “disability”) is added to the respective grade of complication. This label indicates the need for a follow-up to fully evaluate the complication.

^a^Brain hemorrhage, ischemic stroke, subarachnoid hemorrhage, but excluding transient ischemic attacks. *CNS* Central nervous system; *IC* Intermediate care; *ICU* Intensive care unit

### Statistical methods

Data were analyzed using SigmaStat (SigmaStat Version 3.5, Systat Software, Inc., San Jose, CA, USA). For descriptive statistics, data were presented in mean ± standard deviation (SD), unless otherwise stated. Continuous outcomes were compared using Student’s t -test. A *p*-value of < 0.05 was considered statistically significant.

## Results

Fifteen patients underwent selective CSM denervation (9 females and 6 males). All patients were Caucasian and the mean age was 41 ± 10 years. Patients suffered in average from ITNp distributed in the SSd since 13 ± 7 years. Five patients (34%) were smokers and ten (67%) were treated for chronic comorbidities (hypertension, depression, diabetes, etc.).

Medial and lateral motor branches to the CSM, were identified in all patients (Fig. 1). These branches were consistently running within and parallel to the muscle fibers leading to each end of the CSM as previously described in the literature [7–10].

Each patient had utilized nonsteroidal anti-inflammatory drugs (NSAID) and /or triptans, however 80% of them were not consistently responding or had a high rate of side effects (e.g. heartburn, reflux, stomach pain with ulcer formation, dizziness, fatigue, skin rashes) (Table 2).

**Table 2:** Pre- & post-operative drug consumption

Drug	Responder	Non-responder	Responder (sometimes)	Responder (with side effects)
NSAID*	3	–	1	–
Triptans	–	1	–	–
NSAID* + Triptans	–	5	2	3
Tot.	3	6	3	3

*NSAID Nonsteroidal anti-inflammatory drugs

All candidates underwent an average of 3 ± 0 BTA treatments with an interval of 4 ± 1 month. No complications were recorded from BTA injections.

Patients had on average 24 ± 4 migraine days per month (at least one per day), while after surgery this number decreased to 2 ± 2 (*p* < 0.001). The consumption of triptans also decreased significantly (*p* = 0.004) from 11 ± 13 per month to 2 ± 2. The MHI decreased from 208 ± 35 to 10 ± 11(*p* < 0.001).

Four patients (26%) developed ecchymosis of the operative area, which resolved spontaneously within 10 days of surgery. Another patient presented with a small 2a degree burn of the superior palpebral skin (approximately 5 × 5 mm), which healed without visible scar within 12 days. No hematomas, infection, pathologic scaring or injuries to sensory nerves, including temporary loss of sensation were observed. Re-operation and interruption during the procedure due to discomfort were not encountered. According to the Clavien-Dindo complication classification one patient (7%) presented a grade I complication.

CSM function was inhibited immediately after surgery and persisted at the 12-month follow-up visit in all patients. Furthermore, no patient presented any type of animation deformities leading the suspect of a partial CMS re-innervation.

## Discussion

As demonstrated from our data, selective CSM denervation represents a valid treatment tool for patients suffering from ITNp distributed in the SSd, with a low complication rate and a significant reduction of symptoms.

Currently, BTA injections are considered a highly effective and relatively low risk option for decreasing the burden of this type of headaches. However, the main limitation of this approach is the need repeated injections, adding significant costs and inconvenience for patients, and which may not be reimbursed by the health insurance companies. To overcome this limitation, Guyuron first described decompression of the SON and STN with the resection of the medial CSM and fat grafting based on anatomical studies and the hypothesis that these headaches are mainly due to supraorbital and supratrochlear nerve compression [6, 13]. This surgical approach includes several critical steps, usually requiring general anesthesia, including neurolysis and arteriectomy of the supraorbital pedicle, all of which seemed to be critical to the overall outcome [24]. Moreover, resection of the medial head of the corrugator can leave an esthetically unpleasant defect requiring fat grafting [25, 26]. To overcome the limitations of the procedure described by Guyuron, and relying on the positive results of BTA treatments, we aimed to achieve the same BTA effects but permanently via CSM denervation [10]. One of the main criteria for selecting the patients for CSM denervation was an almost complete (near to 100%) remission of ITNp distributed in the SSd after BTA injections. Hypothesizing that the CSM played the most role in the triggering the headaches, our approach did not include a formal neurolysis of the SON or STN pedicles, without release of the supra-orbital notch and arteriectomy. However, CSM denervation in these patients responding to BTA, achieved comparable results to the previously described techniques. This empathizes that the pathophysiology of ITNp distributed in the SSd is still largely unknown, being likely the result of a complex and multifactorial disease rather than then attributable to one main mechanism (nerve compression, vascular pain, muscle hyperactivity, sympathetic hyperactivity, etc.). It is possible in the future that a less invasive and yet more targeted surgical techniques will be used to treat chronic headaches based on the main pain trigger.

Patients suffering from zygomatic-temporal (ZT) triggers can also be treated at the same time as the CSM denervation via the same upper eyelid incision as describe by Hagan et al. In these patients, a lateral dissection under the superficial temporal fascia in the direction of the sentinel vein is carried out using a Trepsat or MUST dissector [27, 28]. The sentinel vein is identified and the ZT nerve is dissected out and avulsed or decompressed without damaging the blood vessel.

Other lesser invasive approaches such as cryotherapy (“frotox”) or targeted radiofrequency ablation have been described in the surgical aesthetic literature to obtain long term denervation of the CSM and may be worth investigating for the treatment of ITNp distributed in the SSd in the future in a subset of patients [29–31]. The surgical technique herein described remains superior in our opinion due to the achievement of permanent results.

Denervation of the CSM also leads to aesthetic improvement due to reduction of frown lines. Aesthetic outcomes can be further improved using the described technique in patients with blepharochalasis by performing an additional upper blepharoplasty via the same approach. In addition, CSM denervation may contribute to the overall improvement in patients suffering also from clinical depression. This hypothesis is suggested by studies demonstrating an improvement of depression after BTA injections [32].

The major limitation of our study is the low number of included patients. In our opinion this fact is partially to conduct to a limited number of patients with chronical headache and migraine seeking for help from a plastic surgeon. Patients with a history of headaches syndromes are normally treated by a high number of different doctors with different treatments and common knowledge does not include that plastic surgeons also treat headache syndromes. Lastly, following the operative treatment all patients clearly presented mimic distortions with a lack of anger and displeasure which the CSM produces. This effect may be appreciated as mentioned by part of the patients, but represents a limitation in more young patients.

## Conclusion

The selective CSM denervation represents a promising, minimally invasive and highly effective treatment option for patients suffering from ITNp distributed in the SSd and responding to BTA injections. Prospective studies with larger numbers of patients are warranted to confirm these initial findings. More knowledge aimed at a better understanding of the complex pathologic mechanisms leading to chronic migraines is needed to develop lesser invasive and more effective approaches.

## Abbreviations

BTA: Botulinum toxin type A
CSM: Corrugator supercilii muscle
ITNp: Idiopatic trigeminal neuralgia purely paroxysmal
MHI: Migraine headache index
MIDAS: Migraine disability assessment score
NSAID: Nonsteroidal anti-inflammatory drugs
SD: Standard deviation
SON: Supraorbital nerve
SSd: Supraorbital and suprathrochlear dermatomes
STN: Supratrochlear nerve
ZT: Zygomatic-temporal
